# Fusion Pores as Regulators of Quantal Size and Cellular Physiology

**DOI:** 10.1002/bies.70064

**Published:** 2025-09-07

**Authors:** Bhavya R. Bhaskar, Shahina Mazumdar, Sruthilaya Dayanandan, Debasis Das

**Affiliations:** ^1^ Department of Biological Sciences Tata Institute of Fundamental Research Mumbai India

## Abstract

The timely release of chemical messengers is a crucial step in cell‐to‐cell communication. Does this release occur as a passive diffusion from the donor membrane or it is actively regulated? A series of studies indicated that chemical messengers’ secretion is “sub‐quantal”. This mode of secretion demands a strongly regulated release mechanism and calls for a thorough characterization of the release sites. When secretory vesicles fuse with the plasma membrane, ephemeral fusion pores serve as the first aqueous connection between the lumen of secretory vesicle and the cell exterior through which chemical messengers are released. Here, we discuss the molecular players that directly regulate fusion pore properties. This has consequences in controlling the amount of chemical messengers’ secretion, hence controlling the quantal size. A thorough understanding of the role of regulatory factors in controlling quantal size can help design potent therapeutics to alter vesicular secretion under pathological conditions.

## Graphical abstract

During regulated exocytosis, membrane fusion regulatory factors act directly on SNAREs and membrane lipids to control fusion pore dynamics. This, in turn, leads to a differential amount of cargo release (referred to in this article as “quantal size”) across various cell types, thereby triggering distinct physiological cues.

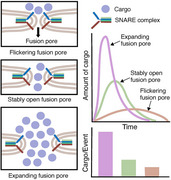

## 1 Introduction

Bernard Katz and colleagues first proposed that the neurotransmitters are released from the presynaptic terminal as discrete “quanta” [1, 2, 3, 4]. They measured postsynaptic responses at the neuromuscular junction as end‐plate potentials (EPPs) using intracellular microelectrode recordings, which allowed them to use EPPs as a sensitive measure of acetylcholine (ACh) release from the presynaptic terminal. Based on those experiments, the synaptic vesicle (SV) discharge from the presynaptic terminal was termed as the quantal event, which occurs in an all‐or‐none manner [1]. According to this proposition, neurotransmitters are packed as discrete quantities of fixed size into a vesicle, which are released completely and randomly from the release sites at the presynaptic terminal and measured as miniature‐EPPs (mEPPs). The mean amplitude of the mEPPs was called the quantal size [1]. When cells are stimulated for secretion, the response is mediated by the release of multiple quanta, because of multiple vesicles’ fusion at the plasma membrane. Thus, the response size varies at random as a random multiple of the quantal size of neurotransmitters is released, which bind to the respective postsynaptic receptors. Several theorists and experimentalists have since worked on improving the initial hypothesis of quantal transmission and posited that a synaptic response per stimulation is a product of the number of vesicle release sites (N), the probability that a vesicle successfully releases its cargo (Pr), and the quantal size [2, 5].

The quantal analysis has historically faced several technical and conceptual challenges due to difficulty in studying a single pre‐ and postsynaptic connection, the effect of noise in intracellular recording that masked the capture of mEPSPs (miniature evoked postsynaptic potential—spontaneous response recorded from postsynapse of neurons), and the signal attenuation and distortion as they propagate along the neuron due to inaccurate postsynaptic recording. These limitations made it difficult to accurately quantify the fundamental parameters of synaptic transmission: N, Pr, and quantal size. Quantal size was believed to be fixed due to experimentally observed regular peaks in amplitude distributions of evoked synaptic currents. However, an early modeling study that simulated evoked response demonstrated that highly nonuniform quantal amplitudes (that arise from varying presynaptic strength) may often overlap to produce regularly spaced evoked amplitude distributions [6].

According to the traditional models of synaptic transmission, the quantal size is governed entirely by postsynaptic factors like receptor number, distribution, and saturation dynamics, with little consideration given to presynaptic variability. This came from the early observation that the amplitude distribution of mEPP/mEPSP varies little from the mean [7, 8, 9, 10, 11], suggesting an apparent saturation of postsynaptic receptors like α‐amino‐3‐hydroxy‐5‐methyl‐4‐isoxazolepropionic acid (AMPA) and *N*‐methyl‐d‐aspartate (NMDA). It was suggested that the quantal size variations observed across neuromuscular junctions and central synapses are primarily due to heterogeneous postsynaptic receptor distribution in these systems. Compelling evidence against this came from a study that showed a large potentiation in AMPA/NMDA currents by exogenously loading glutamate onto the rat brainstem [12]. This study, along with other studies that found large coefficients of variation from the mean quanta [13, 14, 15, 16, 17, 18, 19, 20, 21], suggested that intrinsic variations in glutamate released from a presynaptic bouton were the major determinant of the quantal size. Recent studies using iGluSnFR, a modified glutamate sensor that directly measured the amount of glutamate released from presynapse, showed that there is high variability in the amount of glutamate released from a vesicle and identified it as a source of quantal variability [22]. Currently, it is believed that the quantal size can be affected by the amount of glutamate transported or stored in a single vesicle [21, 23], the size of a vesicle [24, 25, 26], the release kinetics of glutamate through a dynamic fusion pore [21, 23, 27], release of multiple vesicles at a single bouton [28, 29], localization and activity dynamics of postsynaptic receptors [30, 31], and clearance of glutamate from synaptic cleft [16]. Here, we will describe our recent understanding of how vesicle fusion machinery and the fusion pore formed at the site of cargo release affect the quantal size.

The fusion pore is the first aqueous connection formed between the lumen of a secretory vesicle and the extracellular space when secretory vesicles fuse with the plasmalemma during vesicular release. The chemical messengers from different cell types are released through these ephemeral pores in a timely and precise manner. As per the current understanding, soon after the fusion pores are formed, they either close (kiss‐and‐run exocytosis) or vesicles fully fuse with the plasma membrane (full fusion). Kiss‐and‐run mode of fusion would allow for faster vesicle recycling, which would be beneficial in maintaining neurotransmission during high‐frequency stimulation. In this model, the size of the quanta is influenced not only by the amount of neurotransmitter within the vesicle but also by the fusion pore dynamics. This view has evolved partly due to studies in neuroendocrine cells where partial release was easily observed [32, 33, 34, 35], while technical difficulties have imposed limitations on envisaging these pores at the presynaptic terminals of neurons. A series of in vitro biophysical and cell‐based studies have demonstrated the existence of pores and their dynamics in neuroendocrine, endocrine, and immune cells [36, 37, 38, 39]. The high‐resolution amperometry recordings, capacitance measurements, and optical measurements using live cells have demonstrated that fusion pores are highly dynamic and show “flickering” behavior in fast time scales.

Here, we will first focus on the literature that demonstrated the role of fusion pore dynamics in controlling the quantal size during cargo release. As fusion pore behavior remains challenging to characterize in neuronal systems, we include findings from non‐neuronal systems to complement neuronal studies and provide a broader perspective on how fusion pores modulate quantal size and contribute to synaptic plasticity as well as physiological cellular plasticity. This will be followed by the discussion of literature indicating the role of core membrane fusion machinery—soluble *N*‐ethylmaleimide‐sensitive factor attachment protein receptors (SNAREs) and SNARE chaperones in differentially shaping the release site and fusion pore. Overall, in this article, we summarize our current understanding and propose how factors affecting vesicle fusion can account for quantal size variation and their physiological significance.

## 2 Fusion Pore Properties in Determining Quantal Size

**FIGURE 1:**
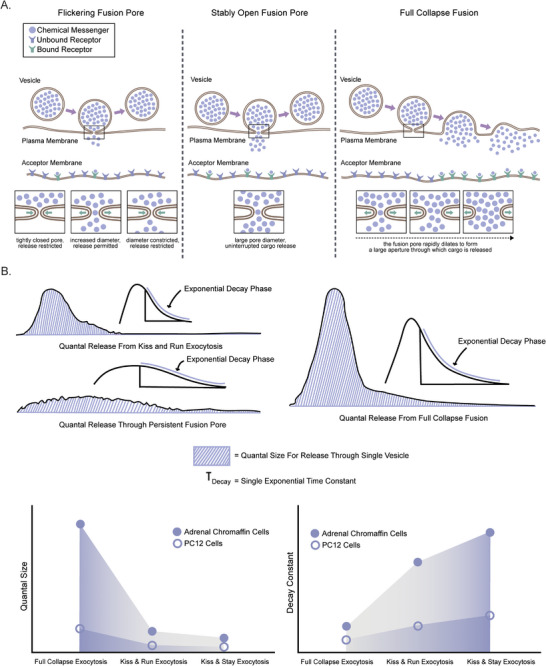
The effect of fusion pore size and dynamics on quantal release. (A) Fusion pore size differs across modes of exocytosis. Cartoon demonstrates three kinetic states of fusion pores – transiently open (indicated as flickering fusion pore), stably open, and fully dilated pore (indicated as full collapse fusion). Insets show enlarged view of different fusion pore states, as indicated. Notably, different kinetic states yield different fusion pore diameters, as indicated. How chemical messengers released through different modes of exocytosis differentially saturate the receptors present in the acceptor membranes is indicated in the cartoon. (B) Amperometric profiles associated with three common modes of exocytosis, depicting the amount of cargo released and the timescale of release events. Adapted from van Kempen et al. [65].

Different modes of exocytosis, such as kiss‐and‐run and full fusion, have been proposed to produce markedly different quantal sizes [40, 41] (Figure 1A). Full collapse fusion releases the bulk of the cargo in one event, while modulation of fusion pore activity during kiss‐and‐run exocytosis through a flickering fusion pore reduces the amount of cargo released per event and draws out the release over longer timescales (Figure 1B). However, the importance of kiss‐and‐run events in explaining neuronal physiology has been debated. One of the reasons for this debate was the idea that there would be less glutamate released if the fusion pore was less stable and thus giving rise to a much slower rise time and/or slow decay in spontaneous and evoked response [42, 43]. Contrary to this view, early capacitance measurements at the calyx of held synapses showed comparable conductance during kiss‐and‐run (>288 pS) and full fusion (>375 pS) [44], with both modes allowing for kinetically fast (sub‐millisecond) release of a large amount of cargo [44] (Figures 1A). Supporting this, an FM dye destaining study correlated the fusion pore behavior and the corresponding miniature evoked postsynaptic current (mEPSC) or action potential‐induced EPSC [43]. They concluded that glutamate release kinetics (and in turn the EPSC decay time) were indeed affected by the fusion pore dynamics and the molecular players involved in vesicle fusion machinery [43]. They found that “kiss and run” mode elicited a slower decay time in mEPSCs. Later, another study using molecular dynamics simulation found that kiss‐and‐run and full fusion can account for both the fastest and slowest fraction of mEPSCs rise time [45], indicating the importance of kiss‐and‐run modes of release in synaptic physiology.

The strength of stimulation can also selectively modulate the release of specific cargo molecules. An optical study using quantum dots differentiated the influence of kiss‐and‐run and full fusion modes depending on high activity demand versus steady state stimulation [46]. They also found the pore open time to be 0.5–1 s, suggesting sub‐second kinetics. Indeed, vesicles of chromaffin cells containing catecholamine and large peptide hormones release norepinephrine in response to weak stimulation, but stronger stimulation triggers the release of both catecholamine and the large peptide chromogranin [47]. When amperometry was used to monitor catecholamine release from mouse chromaffin cells, different kinds of stimuli elicited the exocytosis of similar quantities of neurotransmitter from a large dense‐core vesicle (LDCV) with varying kinetics [48]. Presumably, different stimuli led to the opening of fusion pore with different configurations that in turn bifurcated the response into a slow and small response versus and large and fast response. A short exposure of chromaffin granules with Exendin‐4, a synthetic analog of glucagon‐like peptide‐1 (GLP‐1), increases the quantal size of exocytotic events by switching exocytosis from partial to full fusion [49]. Similarly, the insulin release from pancreatic β‐cell LDCVs required strong stimulation to expand fusion pores [50]. Zhang et al. identified the role of membrane potential in influencing the quantal size of catecholamine release from sympathetic adrenal chromaffin cells, via ATP signaling [51]. This was in accordance with the studies showing that a sustained depolarization increases the content storage within a vesicle [52], whereas an increased frequency of depolarization elevates the quantal size as well as replenishes the readily reusable pool of vesicles [53, 54]. Elhamdani et al. observed that the quantal size increased continuously rather than abruptly with increased stimulation, suggesting that the effect is due to an increase in release efficiency from a single population of vesicles, rather than the recruitment of different‐sized vesicles [55].

In neuroscience, it has long been debated that neurons typically release a single type of neurotransmitter—Dale's principle [56]. However, many findings challenge this notion, revealing that neurons and neuroendocrine cells are capable of co‐releasing multiple neurotransmitters, peptides, or hormones [57, 58, 59]. Interestingly, different cargo stored in the same vesicle shows variable release kinetics through the fusion pore. In one such study, authors used mammalian chromaffin cells to employ a dual‐electrode approach, combining micro‐carbon fiber electrode (CFE) recordings and whole‐cell ATP sniffer recordings [57]. They demonstrated that even though catecholamines and ATP are co‐released from a single vesicle, catecholamine release was sub‐quantal (partial release/kiss‐and‐run mode), whereas ATP release was quantal (full release) [57]. A study on mast cells’ secretory granules, each co‐releasing histamine and 5‐hydroxytryptamine, concluded that cells do not necessarily release chemical messengers in amounts proportional to what they contain [60]. Hence, the amount of cargo discharges (here analogous to the quantal size) can vary, depending upon the fusion pore dynamics, probably through a size exclusion mechanism. Whether the cargo property contributes to the differential fusion pore dynamics requires a detailed investigation.

The relationship between fusion pore behavior and quantal release depends on the amount of cargo packaged into a vesicle, the size of the fusion pore, as well as the amount of time spent by the fusion pore in the open state. A study using genetically modified mice shows that knocking out AP‐3 alters the size of dense core vesicles, reflecting a change in vesicle volume and correspondingly quantal sizes [61]. In another study, altering vesicle loading of catecholamines onto DCVs by using pharmacological modulators alters the quantal size per vesicle but does not reflect in the kinetics of release [62]. Concomitantly, for a distribution of fusion pore sizes across chromaffin cells, the flux through the fusion pore depends on the diameter of the pore, with larger pores linked to a larger quantity of molecules released per unit time [63]. Studies on chromaffin cells show the presence of a direct relationship between pore size and release of cargo through the fusion pore, stating that fusion pores indicated by prespike feet are open for longer timescales and facilitate larger amounts of cargo release [64]. It is also observed that single vesicle release kinetics are much faster for smaller catecholamine cargo [65] as opposed to large hormones such as prolactin [66].

Neurotransmitters are generally packaged into two classes of vesicles, which coexist in most terminals based on their size: small SVs with ∼45 nm diameter and LDCVs with a diameter of <500 nm. Both the above vesicle classes showed a rare kiss‐and‐run mode of fusion when the single vesicle capacitance steps were analyzed using posterior pituitary nerve terminals [42]. The fusion pore conductance through SV was ∼11 times smaller than LDCV [42]. Interestingly, it was found that in the serotonergic Retzius neurons of the leech, the concentration of serotonin stored in SVs and LDCVs was similar but the release of serotonin was slower from LDCVs than from SVs, and the former showed a higher degree of variability [25]. This indicates that fusion pore modulation of different vesicles could determine quantal size since the concentration of cargo stored in each vesicle does not show much variation. Due to technical limitations, fusion pores of small SVs have been difficult to detect, probably due to rapid pore dilation or extremely large initial pores that allowed complete release of neurotransmitters, mimicking full fusion. In neurobiology, the visual demonstration of small‐sized fusion pores in neurons is still lacking and needs to be investigated to further justify the “sub‐quantal release” of neurotransmitters.

Overall, the above studies challenge the quantal model of vesicular secretion, which was proposed to explain neurotransmitter release as discrete packets, or quanta, from SVs. The experimental evidence supports that fusion pore dynamics is critical in controlling quantal size, which dictates the strength of cell‐to‐cell communication in various cell types. The fusion pore dynamics is generally quantified by two parameters – the physical size of the pores and the dwell time of the pore in a certain configuration. Although newer technology is required to probe these two pore properties inside the living cell, recently, techniques like planar bilayer electrophysiology, supported bilayer systems, and optical tools like Total Internal Reflection Fluorescence (TIRF) microscopy have been used to study the fusion pore in vitro [67, 68].

## 3 Approaches to Tracing the Fusion Pores

In studies using non‐neuronal cells, the existence of fusion pores as single, narrow‐necked pores in “quick‐frozen” mast cells was first demonstrated by John E. Heuser using an electron microscope (EM) [69]. The size of the smallest pore was estimated to be ∼50 nm [69]. Freeze‐fractured EM studies on *Tetrahymena pyriformis* suggest the fusion pore aperture widens to ∼245 nm as discharge occurs [70]. In catecholamine‐storing chromaffin cells, release is associated with numerous exo‐endocytotic openings ranging from 20 to 300 nm in diameter [71]. Whereas the patch clamp study using degranulating mast cells demonstrated the initiation of membrane fusion as an aqueous pore of about 230 pS mean conductance, corresponding to a pore diameter of <2 nm [72]. The size of the smallest pore in the same cell type was ∼50 nm, as demonstrated by EM [69], indicating the presence of heterogeneous fusion pore conformations in mast cells. Similarly, electrical recordings in Horse eosinophils, Human neutrophils, Influenza HA, and Semliki forest virus indicated a variation of initial fusion pore conductance between 150 and 300 pS [73, 74, 75, 76]. Patch clamp recording using horse eosinophils [75] and human neutrophils [73] showed initial pore conductance variation from ∼50 to ∼200 pS. It is to be noted that the fusion pore conductance, as measured in all these studies, is an indirect measure of the pore diameter [77, 78]. The above studies using non‐neuronal cells provided evidence that fusion pores can exist in multiple conducting states. The importance of varied fusion pore sizes in cellular physiology warrants further investigation using diverse cell types. The fusion pore structure and composition remain a topic of active debate, whether it is primarily lipidic, proteinaceous, or a combination of both [79, 80, 81, 82]. One hypothesis proposes that the fusion pore initially forms a proteolipidic structure and transitions into a lipidic one during expansion [83], just like the pore formed by the V0 subunit of vacuolar ATPase [84] or the transmembrane rings formed by viral peptide trimers during viral fusion [85]. Since the proteins and lipids lining the fusion pore can significantly influence its stability and, consequently, the quantal size, advanced structural studies are needed to unravel the precise architecture and dynamics of the fusion pore.

**FIGURE 2:**
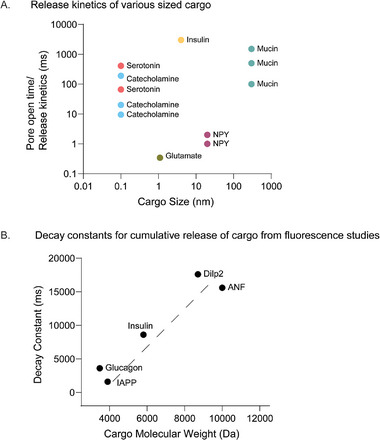
Role of cargo properties in release kinetics. (A) Fusion pore open lifetimes/release kinetics obtained using different techniques (as previously reported) are plotted as a function of cargo size. (B) Time constants for cargo release derived from optical recordings during dense core vesicle (DCV) exocytosis (as reported previously) are plotted as a function of cargo molecular weight.

Different techniques probe different properties of the fusion pore—some measure ion conductance, others measure cargo flux, and others infer membrane rearrangements. For instance, electrophysiological techniques, such as patch‐clamp capacitance measurements or nanodisc–BLM fusion assays, typically interpret pore diameter from electrical conductance using the model of a cylindrical electrolytic pore [86]. This assumption may underestimate actual pore size if the pore is toroidal or surrounded by charged lipids or lined by proteins that restrict ion flow without narrowing the lipidic structure [87]. Moreover, conductance is highly sensitive to ion concentration, pore shape, and access resistance [88], which are not directly measurable in such setups. Thus, electrophysiological methods might underestimate pore sizes due to their assumptions and limitations in spatial resolution [89]. A study utilized super‐resolution STED microscopy to observe dynamic fusion pore behaviors in live neuroendocrine cells, revealing that pore sizes can vary between 0 and 490 nm within milliseconds to seconds [90]. The optical techniques, such as TIRF microscopy or super‐resolution imaging, measure content release or lipid diffusion, unlike conductance measurement in electrophysiology‐based experiments. These methods can cause inaccurate estimation of pore sizes depending on dye size, fluorescent cargo kinetics, and membrane deformation. Therefore, understanding fusion pore dynamics requires integrating across methods and to avoid misinterpreting fusion pore size or stability based on method‐dependent artifacts. Comparing across all methods that studied the release of different cargoes through the fusion pore in systems like chromaffin cells to mast cells, there seems to be no correlation between cargo size and release kinetics (Figure 2A). When comparing across studies that measured cargo release by capturing exocytotic events using only the optical imaging technique led to a positive correlation between cargo size and its release kinetics (Figure 2B). This suggests that we must be aware of the method sensitivity when studying fusion events and cargo release through the fusion pore.

## 4 Molecular Players Assemble Fusion Pores at the Release Sites

**FIGURE 3:**
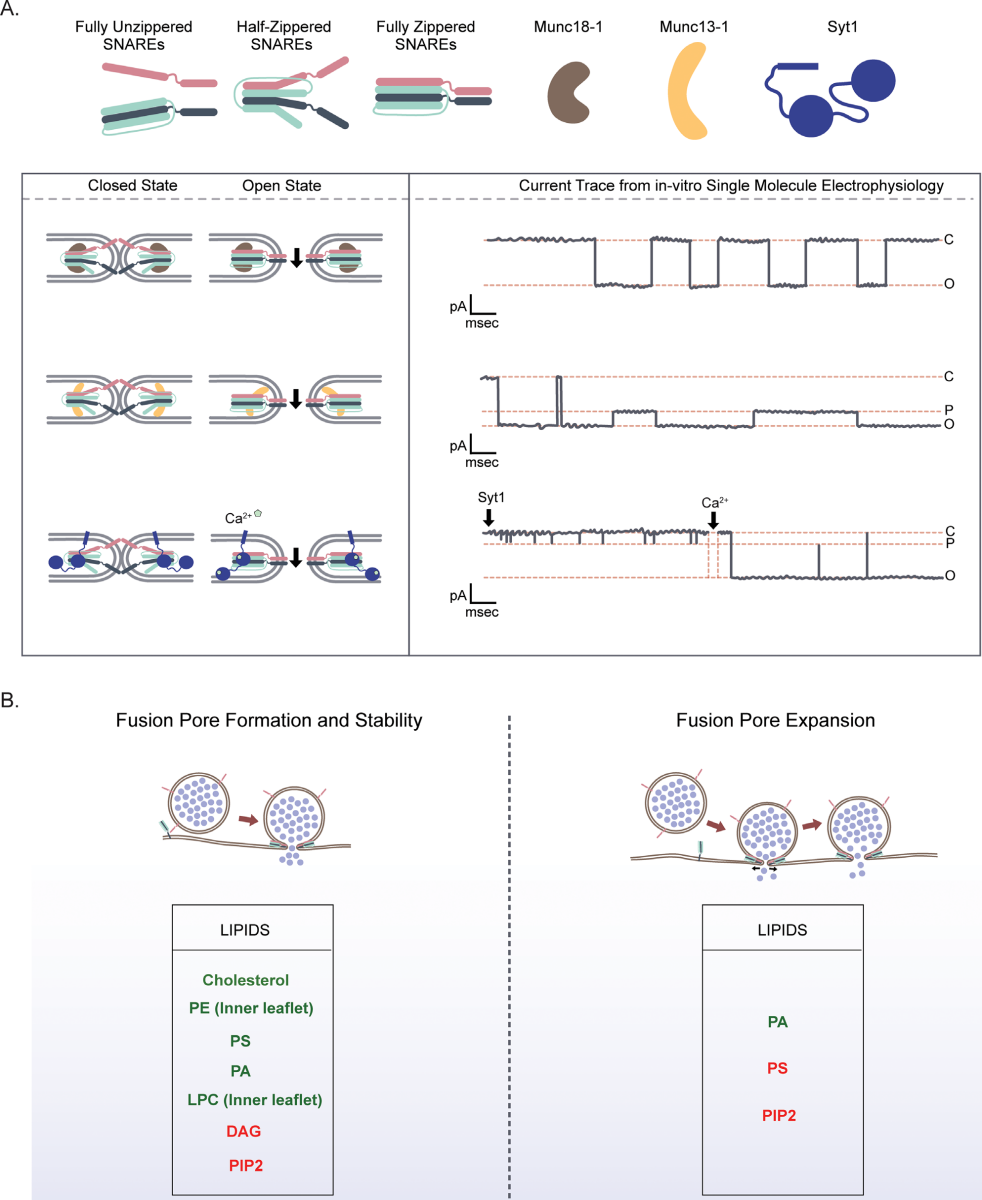
Fusion pore modulation by soluble N‐ethylmaleimide‐sensitive factor attachment protein receptors (SNAREs) and membrane fusion regulatory proteins. (A) Cartoon illustrates how SNAREs, Munc13‐1, Munc18‐1, and Syt1 modulate individual fusion pore properties. The proposed fusion pore structures are shown in the cartoon, with the relevant fusion pore traces obtained from planar bilayer electrophysiology recordings (references [72, 96, 123]); full open (O), partially open (P), and closed (C) states of the pores are indicated. The current (in pA) and time (in millisecond) are mentioned. SNAREs are indicated in pink (Syb2), cyan (SNAP25), dark gray (Syntaxin‐1A) along with their zippering intermediates: unzippered, half‐zippered, and full zippered states; Munc18‐1 is shown in brown, Munc13‐1 is shown in yellow, and Syt1 is shown in violet. (B) Cartoon shows two stages of the fusion pore, pore formation (left) and pore expansion (right), with key regulatory lipids shown at each stage. Lipids that promote the respective stages are marked in green, while those that inhibit are marked in red.

The minimal presynaptic fusion machinery involved in catalysing membrane fusion, leading to the formation of fusion pores, was discovered by James Rothman and colleagues. The minimal machinery involved in releasing chemical messengers from various cell types are SNARE (soluble N‐ethylmaleimide‐sensitive factor attachment protein receptor) proteins [91]. These SNAREs catalyze the fusion of secretory vesicles with the plasma membrane and include t‐SNARE proteins like Syntaxin and SNAP25, and v‐SNARE proteins like Synaptobrevin (Syb2). Currently, we have a reasonable understanding of the release machinery that controls neurotransmitter release from the presynaptic terminal of neurons. The regulated release of neurotransmitters demands a cohort of presynaptic proteins and membrane lipids to act at the release sites. Synaptotagmin 1 (syt1) has been identified as the principal calcium (Ca^2+^) sensor at the presynaptic terminal, whose apo‐configuration significantly reduces release probability before Ca^2+^ influx. Because Ca^2+^ transient sharply rises in a millisecond timescale with shallow decay over a relatively long timeframe, a series of Ca^2+^ sensors have evolved to sense the entire range of intracellular Ca^2+^, like—Synaptotagmin isoforms, Doc2, etc. Another important class of presynaptic regulatory proteins is Munc13‐1 and Munc18‐1, which belong to a class of highly conserved SNARE chaperones [92, 93, 94, 95, 96]. Both the Munc proteins interact with SNAREs and have been shown to template SNARE complexes while protecting them from disassembling factors. Here, we would like to emphasize the role of SNAREs and SNARE chaperones in regulating the fusion pore (Table 1, Figure 3A) and eventually controlling the quantal size.

**TABLE 1:** Cell‐based experiments studying the role of SNARE proteins and SNARE chaperones in modulating fusion pore properties.

Core proteins of the membrane fusion machinery	Cell type	Perturbation‐driven functional change	Effect on the fusion pore	References
Munc18‐1	Adrenal chromaffin cells	Mutant with reduced syntaxin affinity	*Amperometry measurement*: Reduction in rise and fall time of spike (accelerated fusion pore expansion)	[135]
	Lactotroph cells	Mutants with impaired interaction with syntaxin1 and mints	*Capacitance measurement*: Increased time between upward and downward capacitance (increased pore dwell time)	[138]
Munc18‐2	Chromaffin cell	Wild‐type Munc18‐2 (nonendogenously expressed) interacts with native Syntaxin1	*Amperometry measurement*: Fusion pore expansion similar to Munc18‐1	[136]
Munc13‐4	RBL‐2H3 cells	Mutants with altered Ca2+ sensitivity	*TIRF imaging*: Lowering of maximum fluorescence intensity (reduced fusion pore opening) Delayed time to reach peak fluorescent intensity (reduced rate of fusion opening/expansion)	Bin et al., J Immunol. (2018) (PMID: 29884704)
SNAP25	Bovine chromaffin cells	Mutant with decreased tight C‐terminal zippering	*Amperometry measurement*: Decreased PSF and conductance (reduced rate of fusion pore expansion)	[81] Fang et al., J Neurosci. (2015) (PMID: 25698757)
VAMP2/synaptobrevin	Embryonic chromaffin cells	Mutation changing juxtamembrane region length perturbs priming	*Amperometry measurement*: Increased rise time (Impairs fusion pore expansion)	Kesavan et al., Cell (2007) (PMID: 17956735)
VAMP8/endobrevin	Mouse platelets	Absence of VAMP8	*EM analysis*: Average pore diameter at early time point decreased poststimulation (Delayed pore formation) Minimal change in the average pore diameter at later time points (Delayed pore dilation)	Joshi et al., Blood Adv. (2018) (PMID: 30185436)
Syntaxin 1a	PC12 cells	Mutant with reduced acidic phospholipids affinity	*Amperometry measurement*: Increased PSF duration (longer fusion pore duration) Decreased PSF amplitude (smaller pore diameter)	Lam et al., Mol Biol Cell. (2008) (PMID: 18003982)
Synaptotagmin 1	PC12	Over‐expression	*Amperometry measurement*: Increased prespike foot duration (Increased dwell time in narrow configuration before dilation)	[108]
	BON (pancreatic serotonin‐producing neuroendocrine tumor)	Neutralization of highly conserved polybasic patches in either C2 domains	*Amperometry measurement*: Higher spike amplitude with shorter duration (larger fusion pore)	Tsemperouli et al., bioRxiv [Preprint] (2024) (PMID: 39314345)
Synaptotagmin 4	PC12	Overexpression	*Amperometry measurement*: Negligible prespike foot (dilated pore)	Harbord et al., Dev Med Child Neurol. (1990) (PMID: 1691996)

In the kiss‐and‐run mode of vesicle fusion, SNARE complexes are thought to assemble into a ring‐like structure, with at least 5–8 Syntaxin transmembrane domains (TMDs) lining the fusion pore [97] and at most 2 Syb2 molecules [98] to initiate pore nucleation and further pore dilation occurs if more syb2 copies are recruited. A study that perturbed the function of Syb2 found that it not only affected the frequency of mEPSC but also reduced the amplitude and slowed the decay time, indicating a reduction in quantal size [99]. Another study that mutated the Syb2 TMD found a slowing of the rise time of mEPSC recorded from cocultured neurons and HEK cells, indicating the role of fusion pore in regulating quantal size [100]. A study using chromaffin cells found that SNARE complexes can be loosely or tightly formed much before the cells are stimulated, and both states can regulate the initial exocytic burst versus sustained release [101]. Amperometry studies on catecholamine release by Meyer B Jackson's group showed that upon mutating specific amino acid residues of syb2 TMD, the pre‐spike foot amplitude was perturbed [102]. Recently, an in vitro planar bilayer electrophysiology study found that fusion pore size and the kinetic stability of pore open state are regulated by the number of SNAREs present during pore formation [68]. Many studies have found that the efficiency of membrane fusion is affected by the number of SNAREs at the fusion site [103, 104, 105]. It is important to understand whether cells modulate the number of SNARE complexes that constitute a fusion pore and whether this can explain the kiss‐and‐run phenomena and flickering pores observed in cells.

Regulatory proteins that act on SNARE complexes have also been shown to affect quantal release. An earlier study using a CHO cell fusion assay using flipped t‐SNAREs proposed that the binding of regulatory proteins to SNARE complexes may shift the balance between partial release and full fusion, influencing the mode of vesicular release [106]. The calcium sensor Syt1 was found to clamp pore opening and was able to induce a pore dilation only in the presence of calcium [107]. Moreover, amperometry studies on PC12 cells, where two Synaptotagmin isoforms, 1 and 4, were alternatively overexpressed, showed characteristic changes in the pre‐spike foot current amplitude, corroborating that these proteins alter the fusion pore dynamics [108]. A study found that vesicles docked at the center of presynaptic active zones or those that stay for longer timescales at the active zone undergo kiss‐and‐run exocytosis at greater frequencies [109]. These discoveries show that the regulation of the release mechanisms is complex and can be influenced by various factors, including calcium ion sensors, the proteins at the release site, and the overall cellular environment. Hence, for a complete understanding of quantal release, the architecture of the active zone, which includes proteins that facilitate docking and fusion of secretory vesicles with the plasma membrane, must be looked at. As mentioned earlier, two crucial proteins involved in docking and priming the vesicles at the release site are the SNARE chaperones Munc13 and Munc18.

A less‐studied factor in membrane fusion is the membrane lipids. The crucial parameters that lipids tend to regulate are the membrane curvature [110], membrane bending stiffness [111], and membrane line tension [112], all of which play a role in fusion pore opening and stability [113]. Cholesterol has been shown to affect membrane rigidity and is involved in promoting fusion pore opening/expansion [114, 115]. Depletion of cholesterol was also shown to impair exocytosis in neurons [116], as well as affect quantal release and kinetics during subsequent stimulation in chromaffin cells [117]. Mechanistically, cholesterol's ability to induce negative spontaneous curvature and increase fluidity makes the membrane more amenable to both initial bending and later widening of the pore. In contrast, a recent model derived from exocytosis measured from lactotrophs and astrocytes suggests that cholesterol enrichment in vesicles can cause fusion pore constriction [118], which could potentially explain disease conditions like lysosomal storage diseases, where cholesterol accumulates in vesicles. Regardless of whether cholesterol positively or negatively regulates fusion pore dynamics, it seems to have a role in pore modulation. Similarly, other lipids like phosphatidyl inositol, phosphatidyl serine, phosphatidic acid, and diacylglycerol (DAG) have been shown to affect membrane fusion dynamics and fusion pore properties [119, 120, 121, 122, 123, 124, 125, 126, 127] (Figure 3B). Many core proteins involved in membrane fusion are known to bind to lipids like PS, PI(4,5)P2, and DAG [128, 129, 130], which also need to be considered while studying how these proteins and lipids act in concert to regulate fusion pore properties (Figure 3).

## 5 Functional Heterogeneity of SNARE Chaperones – Source of Quantal Size Variation?

SNARE chaperones are a class of highly conserved proteins that work in a concerted manner to orchestrate the process of vesicle fusion [131]. Munc18 is reported to possess two contradictory functions—preventing SNARE complex assembly via maintaining a “closed” conformation of the target membrane SNARE Syntaxin [132], and enhancing SNARE complex assembly by templating directional SNARE zippering [133]. The directionality of zippering is essential for SNAREs, as the propensity of coiled‐coil domain formation is extremely high and can lead to off‐pathway zippered products in the absence of regulators. Munc18‐mediated closure of Syntaxin prevents indiscriminate fusion. Another chaperone that “opens” closed Syntaxin to form proper SNARE complexes is Munc13. Munc13 contains domains for binding to Ca^2+^, membrane lipids such as PI(4,5)P2, DAG, PS, and other regulatory proteins. Two regulatory conformations of this Munc protein were hypothesized, the first being a rigid straight conformation, which prevents vesicle fusion, and the second being an oblique conformation, which enables membrane binding and vesicle bridging by bringing the donor and acceptor membranes closer [134]. The two chaperones thus work cooperatively to enable SNARE zippering, which provides the mechanical force to pull the vesicular and target membranes closer for the formation of a continuous aqueous passage between the two membranes. It was, however, unclear if these Munc proteins stay at the release site after the fusion pore opening and possess any regulatory role in controlling pore characteristics.

A Munc18 mutant with reduced affinity for Syntaxin was found to decrease quantal size while accelerating the opening of the initial fusion pore, which subsequently reclosed, indicative of a kiss‐and‐run mechanism [135]. This suggests that dissociation of Munc18 from Syntaxin can influence postfusion pore dynamics. However, a subsequent study contradicted these findings, reporting that Munc18 does not directly regulate the fusion pore but instead plays a role upstream in vesicle docking and priming [136]. Other research has proposed a Munc18‐Syntaxin interaction‐independent pathway for pore modulation, involving interactions between Munc18 and non‐SNARE proteins such as Mint and Rab3a [137, 138]. Mutations in Munc18 that disrupted these interactions were observed to stabilize narrow fusion pores while enhancing cargo release, possibly by preferentially facilitating the fusion of larger vesicles. Nevertheless, the possibility that these mutations also interfered directly with SNARE complex assembly, independently of Munc18's interactions with other regulatory proteins, cannot be ruled out. Because Munc18‐1 and Syntaxin‐1 conformations alter during neurotransmitter release [139, 140], the modulation of the Munc18‐1/Syntaxin‐1 interaction would play a crucial role in determining quantal size and release kinetics. The findings from several studies collectively highlight the connection between the strength of Munc18‐1/Syntaxin‐1 interaction and the efficiency of cargo release [141, 142, 143, 144]. Graham et al. [145] found that the increased catecholamine release from adrenal chromaffin cells upon Syntaxin 1a mutant overexpression was due to its impaired interaction with Munc18‐1, rather than altered vesicle loading. Munc18‐1 was previously shown to regulate catecholamine release from individual granules. The expression of a mutant Munc18 (R39C) led to a 45% reduction in total charge per spike—a measure of how much catecholamine is released. Furthermore, this reduction in total charge correlated with a decrease in half‐width as well as a decrease in both rise times and fall times for spikes [135]. This suggests that increased Munc18‐1 levels may act as a critical modulator of both the efficiency and timing of hormone release.

Though Munc13‐1 is extremely critical for secretory vesicle fusion, few studies have looked at its role in controlling fusion pore behavior. Indirect evidence of its importance was observed, which found the role of phorbol esters mimic in affecting quantal release. This is particularly interesting because Munc13‐1 has a C1 domain that binds to the phorbol ester DAG. An amperometry study looking at dopaminergic release in rat ventral midbrain found that PDBu treatment decreased the flickering of the fusion pore [146]. Similarly, another phorbol ester, PMA, has also been shown to affect fusion pore kinetics [147]. Although some studies suggested that protein kinase C (PKC) is the molecular target of phorbol esters to enhance secretion, many others suggested that Munc13 plays a major role in regulating phorbol ester‐mediated enhanced release [148, 149, 150]. Additionally, many PKC inhibitors were unable to block phorbol ester‐mediated increased secretion [151, 152], strengthening the importance of Munc13 in mediating the increased synaptic efficacy by phorbol esters. Another study in neurons found that inhibiting Munc13 function using a peptide reduced the phorbol ester's effect on secretion [153].

A direct action of Munc13‐1 and Munc18‐1 on fusion pore formation was studied recently using in vitro planar bilayer electrophysiology, where it was found that differential action of the SNARE chaperones Munc13‐1 and Munc18‐1 allowed for variable configurations of the fusion pore in terms of both the size as well as pore opening kinetics [124] (Figure 3A). This study indicated that DAG‐bound Munc13 restricts the fusion pore to a narrow structure but a kinetically stable open state. Munc18, on the other hand, opened the pore into a physically large size but with kinetically unstable in open states. Variable pore size and kinetic stability shown by fusion pores in the presence of these SNARE chaperones indicate how the complex cellular environment and molecular players play an important role in regulating pore behavior. It remains to be seen how this translates into variability in quantal sizes seen in most secretory cell types.

## 6 Is Quantal Size Variation Important for Cellular Physiology?

**FIGURE 4:**
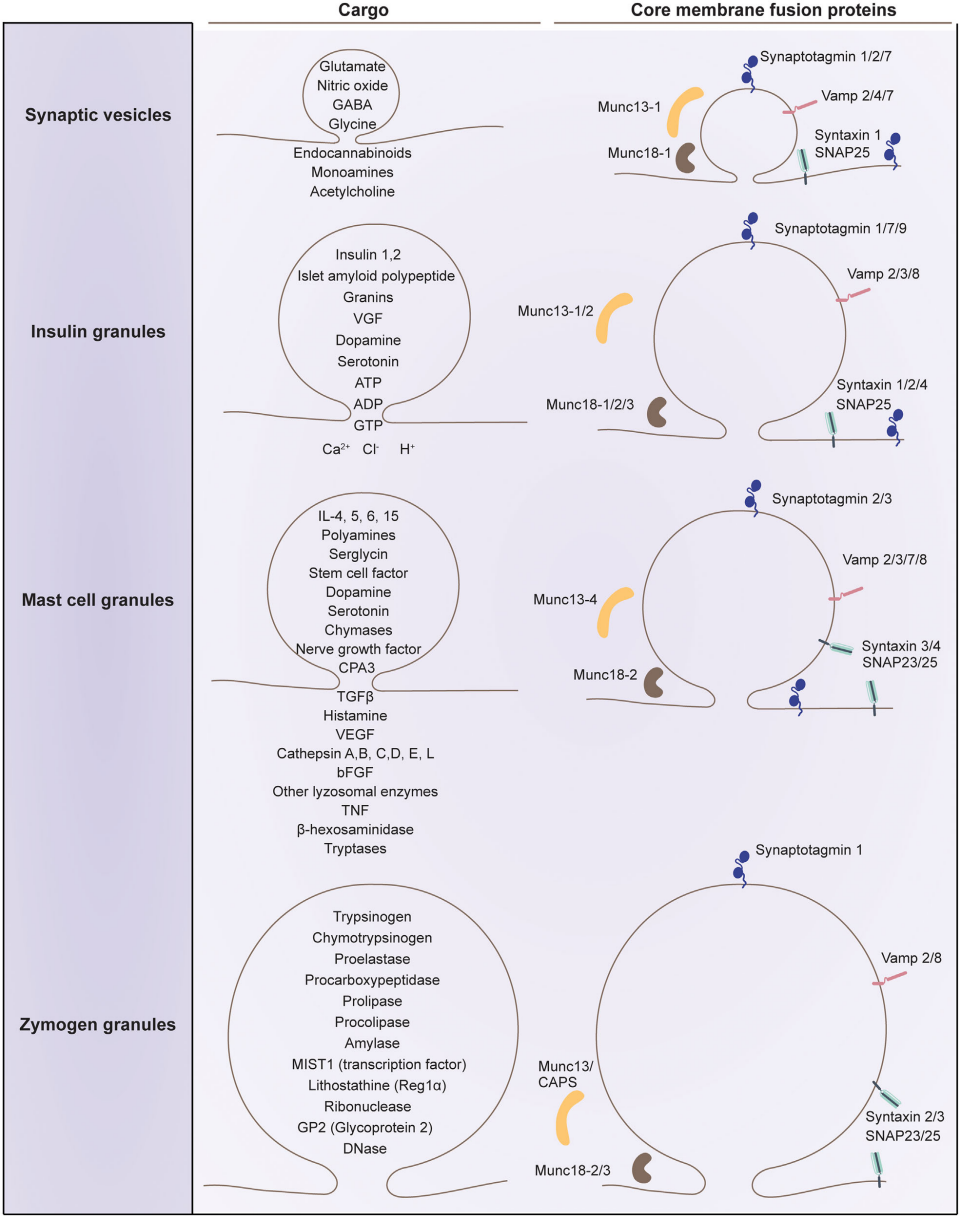
Heterogeneity in vesicle size, cargo properties, and core membrane fusion proteins across various secretion machinery. The cartoon depicts four types of secretory vesicles—synaptic vesicles, insulin granules, mast cell granules, and zymogen granules, illustrating their relative sizes, cargo contents, and associated soluble N‐ethylmaleimide‐sensitive factor attachment protein receptor (SNARE) fusion machinery.

A critical question in cellular physiology is whether the dynamic flickering of fusion pores has functional significance. Few studies have explored the physiological relevance of “sub‐quantal” release, which arises from variations in fusion pore dynamics. The fusion pore conductance and temporal dynamics, its ability to dilate or reseal, act as a physical gatekeeper for quantal release, shaping not only the amount of vesicular content released (quantal size) but also the temporal profile of that release. In neurons, while mEPP/mEPSPs from which quantal size is derived are believed to be noise that influences synaptic strength and plasticity [154], in non‐neuronal cells, such as endocrine, immune, or epithelial cells, the variability in quantal size and timing is more accepted to be a functionally tuned parameter [47, 155]. For instance, in chromaffin cells, basal sympathetic firing facilitates the release of catecholamines, while chronic stimulation enables the co‐release of catecholamines and neuropeptides from the same vesicle [47]. This suggests that the “fight or flight” response of the sympathetic system may be mediated by a regulated, activity‐dependent dilation of the fusion pore and a size‐exclusion mechanism that governs differential cargo release [47]. In immune cells, such as mast cells or cytotoxic T cells, transient fusion pore openings may allow release of only a subset of granular content (e.g., small chemokines but not large proteases), thus modulating the immune response based on context [155]. From a computational perspective, partial release via transient or narrow pores allows cells to encode graded outputs from an all‐or‐none vesicle fusion event. This allows for fine‐tuning of cellular responses to external stimuli, such as adjusting the amount of insulin secreted in response to glucose levels or modulating cytokine release in immune signaling. The fusion pore provides an analog interface, allowing the cell to integrate environmental signals and produce nuanced, temporally distributed outputs while considering energetic demands. The localized lipid composition and heterogeneous protein distribution create a rich spatiotemporal code for vesicle fusion events in both neuronal and non‐neuronal systems (Figure 4).

In endocrine and exocrine cells, where signaling often occurs over greater distances than in paracrine or autocrine systems, hormones released from the source cell must traverse the circulation to reach their targets. A fully dilated, long‐lived fusion pore may release a large bolus of hormones rapidly, while transient or narrow pores could limit the diffusion of certain macromolecules, functioning much like a valve rather than a simple on‐off switch. This raises questions about the physiological impact of quantal size variation in hormone release. The availability of hormones to target cells depends not only on the frequency and amplitude of their release but also on factors such as transport, clearance, and bioavailability in the circulation. Since the frequency of hormone pulses is critical for their physiological effects [156, 157], it is essential to investigate whether variations in quantal size (and the effect of fusion pore dynamics) during release contribute to this pulsatile secretion.

Unlike synaptic transmission, another example of short‐distance communication between cells, where the released chemical messengers from one cell rapidly diffuse and interact with the neighboring cells, is in the case of morphogen gradients during morphogenesis and cell fate determination. Several factors are crucial for the morphogen gradient formation, for example, extracellular diffusion, receptor binding, internalization, recycling, and intra‐ and extracellular degradation. The morphogens are secreted from the source cells through exocytosis [158]. The maintenance of a strict morphogen gradient requires a tightly regulated release of morphogens from the source cells. How the source cells’ release machinery controls the quantal size of morphogens is still unclear. Future studies are required to characterize the fusion pore assembly involved in morphogen secretion. Evidence for the importance of exocytotic machinery in maintaining the morphogen gradient was shown during the development of the *Drosophila* air sac primordia [159]. A partial loss of function of Synaptobrevin and Synaptotagmin in the wing disk cells, which affected the release of a signaling morphogen called dpp (a bone morphogenetic protein), caused a smaller air sac primordium to be formed.

In neuronal communication, substantial research has focused on understanding the molecular basis of neuronal plasticity, the cellular mechanism proposed to underlie learning and memory. Synaptic responses are finely tuned to enable brain networks to adapt to environmental changes. During repetitive neuronal activity, synaptic strength can transiently increase (facilitation) or decrease (depression), phenomena collectively termed short‐term synaptic plasticity. This modulation is influenced by several pre‐ and postsynaptic factors, including the availability of distinct vesicle pools at the presynapse, presynaptic calcium signaling, and postsynaptic receptor expression. During short‐term synaptic plasticity at single hippocampal synapses, paired‐pulse facilitation was shown to result from an increase in release probability, while paired‐pulse depression was driven by a decrease in quantal size, mediated through fusion pore modulation [160]. Additionally, presynaptic components of long‐term potentiation (LTP) and long‐term depression (LTD) have been attributed to changes in release probability and the silencing of presynaptic terminals [15, 161, 162]. In the CA1 region of the mammalian hippocampus, a critical site for memory modulation, LTP and LTD have been associated with increases and decreases in quantal size, respectively [163, 164]. In dopaminergic neurons, dopamine release occurs via a flickering fusion pore, which can be influenced by phorbol esters and staurosporine [146]. Similarly, fusion pore kinetics and presynaptic fusion modes play crucial roles in the maturation of silent synapses into functional ones [162, 165, 166]. Studies have demonstrated that differential fusion pore‐mediated glutamate release affects postsynaptic receptor activation, with slow release via kiss‐and‐run desensitizing AMPA receptors, whereas full fusion‐mediated release robustly activates NMDA receptors [165]. Interestingly, they found that disrupting SNARE‐mediated fusion with tetanus toxin can revert functional synapses to a silent state. At the neuromuscular junction in the diaphragm, where neuronal discharges stimulate muscle contraction for respiration, both kiss‐and‐run and full‐fusion modes of release have been observed. Under stress, a switch from full fusion to kiss‐and‐run prevents overstimulation of the respiratory system, highlighting a regulatory mechanism for maintaining respiratory homeostasis [167]. A de novo dominant mutant of SNAP25B was reported, which caused a complex neurological disorder characterized by congenital myasthenia, cortical hyperexcitability, cerebellar ataxia, and intellectual disability, significantly impaired quantal release at the neuromuscular junction [168].

Overall, studies using different cell types indicated the importance of quantal size variation in cellular physiology. These studies collectively underscore the critical role of fusion pore dynamics in regulating cargo release, with significant implications for downstream signaling and functional adaptation in several secretory cells, including those of the neuronal and neuromuscular systems (Figure 4).

## 7 Conclusions

Because the variable quantal size differentially affects cellular physiology, it is crucial to thoroughly investigate the factors responsible for quantal size variation in different cell and tissue types. This variation can result in pathological consequences. Fusion pore dynamics is one such factor that can be regulated to allow for use‐dependent changes in quantal size. The mode of action of the cellular factors, like Munc13 and Munc18 as described in this article, will be crucial to design potent therapeutics, to pharmacologically alter the fusion pore, and thus, the quantal size.

## Author Contributions

D.D. and B.R.B conceptualized. B.R.B., S.M., S.D., and D.D. wrote the manuscript.

## Conflicts of Interest

The authors declare no conflicts of interest.

## Data Availability

The data that support the findings of this study are available from the corresponding author upon reasonable request.

## References

[1] P. Fatt and B. Katz , “Spontaneous Subthreshold Activity at Motor Nerve Endings,” The Journal of Physiology 117 (1952): 109–128. 14946732 PMC1392564

[2] J. Del Castillo and B. Katz , “Quantal Components of the End‐Plate Potential,” The Journal of Physiology 124 (1954): 560–573, 10.1113/jphysiol.1954.sp005129. 13175199 PMC1366292

[3] M. Kuno , “Quantal Components of Excitatory Synaptic Potentials in Spinal Motoneurones,” The Journal of Physiology 175 (1964): 81–99, 10.1113/jphysiol.1964.sp007504. 14241159 PMC1357086

[4] P. Fatt and B. Katz , “An Analysis of the End‐Plate Potential Recorded With an Intra‐Cellular Electrode,” The Journal of Physiology 115 (1951): 320–370, 10.1113/jphysiol.1951.sp004675. 14898516 PMC1392060

[5] V. Scheuss and E. Neher , “Estimating Synaptic Parameters From Mean, Variance, and Covariance in Trains of Synaptic Responses,” Biophysical Journal 81 (2001): 1970–1989, 10.1016/S0006-3495(01)75848-1. 11566771 PMC1301672

[6] B. Walmsley , “Interpretation of ‘Quantal’ Peaks in Distributions of Evoked Synaptic Transmission at Central Synapses,” Proceedings of the Royal Society 261 (1995): 245–250, 10.1098/rspb.1995.0144. 7568277

[7] J. J. Jack , S. J. Redman , and K. Wong , “The Components of Synaptic Potentials Evoked in Cat Spinal Motoneurones by Impulses in Single Group Ia Afferents,” The Journal of Physiology 321 (1981): 65–96. 6279826 10.1113/jphysiol.1981.sp013972 PMC1249614

[8] M. Frerking and M. Wilson , “Saturation of Postsynaptic Receptors at Central Synapses?” Current Opinion in Neurobiology 6 (1996): 395–403, 10.1016/s0959-4388(96)80125-5. 8794082

[9] S. Redman , “Quantal Analysis of Synaptic Potentials in Neurons of the Central Nervous System,” Physiological Reviews 70 (1990): 165–198, 10.1152/physrev.1990.70.1.165. 2404288

[10] J. Kleinle , K. Vogt , H. R. Lüscher , et al., “Transmitter Concentration Profiles in the Synaptic Cleft: An Analytical Model of Release and Diffusion,” Biophysical Journal 71 (1996): 2413–2426, 10.1016/S0006-3495(96)79435-3. 8913582 PMC1233731

[11] M. Frerking and M. Wilson , “Differences in Uniquantal Amplitude Between Sites Reduce Uniquantal Variance When Few Release Sites Are Active,” Synapse 32 (1999): 276–287, 10.1002/(SICI)1098-2396(19990615)32:4<276::AID-SYN4>3.0.CO;2-3. 10332803

[12] T. Ishikawa , Y. Sahara , and T. Takahashi , “A Single Packet of Transmitter Does Not Saturate Postsynaptic Glutamate Receptors,” Neuron 34 (2002): 613–621, 10.1016/s0896-6273(02)00692-x. 12062044

[13] T. Yamashita , T. Ishikawa , and T. Takahashi , “Developmental Increase in Vesicular Glutamate Content Does Not Cause Saturation of AMPA Receptors at the Calyx of Held Synapse,” The Journal of Neuroscience 23 (2003): 3633–3638, 10.1523/JNEUROSCI.23-09-03633.2003. 12736334 PMC6742176

[14] A. K. McAllister and C. F. Stevens , “Nonsaturation of AMPA and NMDA Receptors at Hippocampal Synapses,” Proceedings of the National Academy of Sciences *of the United States of America* 97 (2000): 6173–6178, 10.1073/pnas.100126497. PMC18577 10811899

[15] J. M. Bekkers and C. F. Stevens , “Presynaptic Mechanism for Long‐Term Potentiation in the Hippocampus,” Nature 346 (1990): 724–729, 10.1038/346724a0. 2167454

[16] W. R. Holmes , “Modeling the Effect of Glutamate Diffusion and Uptake on NMDA and Non‐NMDA Receptor Saturation,” Biophysical Journal 69 (1995): 1734–1747, 10.1016/S0006-3495(95)80043-3. 8580317 PMC1236407

[17] J. N. Johannessen and S. P. Markey , “Assessment of the Opiate Properties of Two Constituents of a Toxic Illicit Drug Mixture,” Drug and Alcohol Dependence 13 (1984): 367–374, 10.1016/0376-8716(84)90004-8. 6148225

[18] Z. F. Mainen , R. Malinow , and K. Svoboda , “Synaptic Calcium Transients in Single Spines Indicate that NMDA Receptors Are Not Saturated,” Nature 399 (1999): 151–155, 10.1038/20187. 10335844

[19] J. C. Behrends and G. ten Bruggencate , “Changes in Quantal Size Distributions Upon Experimental Variations in the Probability of Release at Striatal Inhibitory Synapses,” Journal of Neurophysiology 79 (1998): 2999–3011, 10.1152/jn.1998.79.6.2999. 9636103

[20] L. Forti , M. Bossi , A. Bergamaschi , A. Villa , and A. Malgaroli , “Loose‐Patch Recordings of Single Quanta at Individual Hippocampal Synapses,” Nature 388 (1997): 874–878, 10.1038/42251. 9278048

[21] E. Hanse and B. Gustafsson , “Quantal Variability at Glutamatergic Synapses in Area CA1 of the Rat Neonatal Hippocampus,” The Journal of Physiology 531 (2001): 467–480, 10.1111/j.1469-7793.2001.0467i.x. 11230519 PMC2278484

[22] C. Soares , D. Trotter , A. Longtin , J.‐C. Béïque , and R. Naud , “Parsing Out the Variability of Transmission at Central Synapses Using Optical Quantal Analysis,” Frontiers in Synaptic Neuroscience 11 (2019): 22, 10.3389/fnsyn.2019.00022. 31474847 PMC6702664

[23] D. Engel , I. Pahner , K. Schulze , et al., “Plasticity of Rat Central Inhibitory Synapses Through GABA Metabolism,” The Journal of Physiology 535 (2001): 473–482, 10.1111/j.1469-7793.2001.00473.x. 11533137 PMC2278801

[24] S. Karunanithi , L. Marin , K. Wong , and H. L. Atwood , “Quantal Size and Variation Determined by Vesicle Size in Normal and Mutant *Drosophila* Glutamatergic Synapses,” The Journal of Neuroscience 22 (2002): 10267–10276, 10.1523/JNEUROSCI.22-23-10267.2002. 12451127 PMC6758758

[25] D. Bruns , D. Riedel , J. Klingauf , and R. Jahn , “Quantal Release of Serotonin,” Neuron 28 (2000): 205–220, 10.1016/s0896-6273(00)00097-0. 11086995

[26] J. R. Steinert , H. Kuromi , A. Hellwig , et al., “Experience‐Dependent Formation and Recruitment of Large Vesicles From Reserve Pool,” Neuron 50 (2006): 723–733, 10.1016/j.neuron.2006.04.025. 16731511

[27] X.‐S. Wu , L. Xue , R. Mohan , K. Paradiso , K. D. Gillis , and L.‐G. Wu , “The Origin of Quantal Size Variation: Vesicular Glutamate Concentration Plays a Significant Role,” The Journal of Neuroscience 27 (2007): 3046–3056, 10.1523/JNEUROSCI.4415-06.2007. 17360928 PMC6672571

[28] J. M. Bekkers , G. B. Richerson , and C. F. Stevens , “Origin of Variability in Quantal Size in Cultured Hippocampal Neurons and Hippocampal Slices,” Proceedings of the National Academy of Sciences *of the United States of America* 87 (1990): 5359–5362, 10.1073/pnas.87.14.5359. PMC54323 2371276

[29] S. Raghavachari and J. E. Lisman , “Properties of Quantal Transmission at CA1 Synapses,” Journal of Neurophysiology 92 (2004): 2456–2467, 10.1152/jn.00258.2004. 15115789

[30] Z. Nusser , S. Cull‐Candy , and M. Farrant , “Differences in Synaptic GABAA Receptor Number Underlie Variation in GABA Mini Amplitude,” Neuron 19 (1997): 697–709, 10.1016/s0896-6273(00)80382-7. 9331359

[31] S. Oleskevich , F. J. Alvarez , and B. Walmsley , “Glycinergic Miniature Synaptic Currents and Receptor Cluster Sizes Differ Between Spinal Cord Interneurons,” Journal of Neurophysiology 82 (1999): 312–319, 10.1152/jn.1999.82.1.312. 10400960

[32] J. M. Fernandez , E. Neher , and B. D. Gomperts , “Capacitance Measurements Reveal Stepwise Fusion Events in Degranulating Mast Cells,” Nature 312 (1984): 453–455, 10.1038/312453a0. 6504157

[33] G. A. De Toledo , R. Fernández‐Chacón , and J. M. Fernández , “Release of Secretory Products During Transient Vesicle Fusion,” Nature 363 (1993): 554–558, 10.1038/363554a0. 8505984

[34] E. Alés , L. Tabares , J. M. Poyato , V. Valero , M. Lindau , and G. Alvarez De Toledo , “High Calcium Concentrations Shift the Mode of Exocytosis to the Kiss‐and‐Run Mechanism,” Nature Cell Biology 1 (1999): 40–44, 10.1038/9012. 10559862

[35] J. Cheng and M. B. Jackson , “Somatostatin Modulation of Initial Fusion Pores in Ca 2+‐Triggered Exocytosis From Mouse Chromaffin Cells,” The Journal of Physiology (2024):, 10.1113/JP286175. PMC11825891 39141801

[36] J. M. Cabeza , J. Acosta , and E. Alés , “Mechanisms of Granule Membrane Recapture Following Exocytosis in Intact Mast Cells,” Journal of Biological Chemistry 288 (2013): 20293–20305, 10.1074/jbc.M113.459065. 23709219 PMC3711296

[37] A. F. Oberhauser and J. M. Fernandez , “A Fusion Pore Phenotype in Mast Cells of the Ruby‐Eye Mouse,” Proceedings of the National Academy of Sciences *of the United States of America* 93 (1996): 14349–14354, 10.1073/pnas.93.25.14349. PMC26135 8962054

[38] L. Ge , W. Shin , G. Arpino , et al., “Sequential Compound Fusion and Kiss‐and‐Run Mediate Exo‐ and Endocytosis in Excitable Cells,” Science Advances 8 (2022): abm6049, 10.1126/sciadv.abm6049. PMC9205584 35714180

[39] S. A. Chaney and D. Thomas , “Restoration of Abutment Teeth for an Existing Removable Partial Denture,” Journal (Canadian Dental Association) 47 (1981): 115–117. 7011501

[40] A. A. Alabi and R. W. Tsien , “Perspectives on Kiss‐and‐Run: Role in Exocytosis, Endocytosis, and Neurotransmission,” Annual Review of Physiology 75 (2013): 393–422, 10.1146/annurev-physiol-020911-153305. 23245563

[41] L. G. Wu , E. Hamid , W. Shin , and H. C. Chiang , “Exocytosis and Endocytosis: Modes, Functions, and Coupling Mechanisms,” Annual Review of Physiology 76 (2014): 301–331, 10.1146/annurev-physiol-021113-170305. PMC4880020 24274740

[42] V. A. Klyachko and M. B. Jackson , “Capacitance Steps and Fusion Pores of Small and Large‐Dense‐Core Vesicles in Nerve Terminals,” Nature 418 (2002): 89–92, 10.1038/nature00852. 12097912

[43] C. Pawlu , A. DiAntonio , and M. Heckmann , “Postfusional Control of Quantal Current Shape,” Neuron 42 (2004): 607–618, 10.1016/s0896-6273(04)00269-7. 15157422

[44] L. He , X. S. Wu , R. Mohan , and L. G. Wu , “Two Modes of Fusion Pore Opening Revealed by Cell‐Attached Recordings at a Synapse,” Nature 444 (2006): 102–105, 10.1038/nature05250. 17065984

[45] J. Guo , Z. C. Sun , P. T. Yao , H. L. Wang , and L. Xue , “A Monte Carlo Simulation Dissecting Quantal Release at the Calyx of Held,” Frontiers in Bioscience (Landmark Ed) 20 (2015): 1079–1091, 10.2741/4360. 25961546

[46] Q. Zhang , Y. Li , and R. W. Tsien , “The Dynamic Control of Kiss‐and‐Run and Vesicular Reuse Probed With Single Nanoparticles,” Science 323 (2009): 1448–1453, 10.1126/science.1167373. 19213879 PMC2696197

[47] T. Fulop , S. Radabaugh , and C. Smith , “Activity‐Dependent Differential Transmitter Release in Mouse Adrenal Chromaffin Cells,” The Journal of Neuroscience 25 (2005): 7324–7332, 10.1523/JNEUROSCI.2042-05.2005. 16093382 PMC6725304

[48] C. P. Grabner and A. P. Fox , “Stimulus‐Dependent Alterations in Quantal Neurotransmitter Release,” Journal of Neurophysiology 96 (2006): 3082–3087, 10.1152/jn.00017.2006. 16956996

[49] A. González‐Santana , J. Estévez‐Herrera , E. P. Seward , R. Borges , and J. D. Machado , “Glucagon‐Like Peptide‐1 Receptor Controls Exocytosis in Chromaffin Cells by Increasing Full‐Fusion Events,” Cell Reports 36 (2021): 109609, 10.1016/j.celrep.2021.109609. 34433018

[50] P. E. MacDonald , M. Braun , J. Galvanovskis , and P. Rorsman , “Release of Small Transmitters Through Kiss‐and‐Run Fusion Pores in Rat Pancreatic β Cells,” Cell Metabolism 4 (2006): 283–290, 10.1016/j.cmet.2006.08.011. 17011501

[51] Q. Zhang , B. Liu , Y. Li , et al., “Regulating Quantal Size of Neurotransmitter Release Through a GPCR Voltage Sensor,” Proceedings of the National Academy of Sciences *of the United States of America* 117 (2020): 26985–26995, 10.1073/pnas.2005274117. PMC7604499 33046653

[52] D. G. Bole , K. Hirata , and T. Ueda , “Prolonged Depolarization of Rat Cerebral Synaptosomes Leads to an Increase in Vesicular Glutamate Content,” Neuroscience Letters 322 (2002): 17–20, 10.1016/s0304-3940(02)00105-2. 11958833

[53] L. Y. Wang and L. K. Kaczmarek , “High‐Frequency Firing Helps Replenish the Readily Releasable Pool of Synaptic Vesicles,” Nature 394 (1998): 384–388, 10.1038/28645. 9690475

[54] D. Li , Y. Zhu , and H. Huang , “Spike Activity Regulates Vesicle Filling at a Glutamatergic Synapse,” The Journal of Neuroscience 40 (2020): 4972–4980, 10.1523/JNEUROSCI.2945-19.2020. 32430294 PMC7314407

[55] A. Elhamdani , H. C. Palfrey , and C. R. Artalejo , “Quantal Size Is Dependent on Stimulation Frequency and Calcium Entry in Calf Chromaffin Cells,” Neuron 31 (2001): 819–830, 10.1016/s0896-6273(01)00418-4. 11567619

[56] V. J. Barranca , A. Bhuiyan , M. Sundgren , and F. Xing , “Functional Implications of Dale's Law in Balanced Neuronal Network Dynamics and Decision Making,” Frontiers in Neuroscience 16 (2022): 801847, 10.3389/fnins.2022.801847. 35295091 PMC8919085

[57] Q. Zhang , B. Liu , Q. Wu , et al., “Differential Co‐Release of Two Neurotransmitters From a Vesicle Fusion Pore in Mammalian Adrenal Chromaffin Cells,” Neuron 102 (2019): 173–183.e4, 10.1016/j.neuron.2019.01.031. 30773347

[58] C. E. Vaaga , M. Borisovska , and G. L. Westbrook , “Dual‐Transmitter Neurons: Functional Implications of Co‐Release and Co‐Transmission,” Current Opinion in Neurobiology 29 (2014): 25–32, 10.1016/j.conb.2014.04.010. 24816154 PMC4231002

[59] I. Merchenthaler , F. J. Lopez , and A. Negro‐Vilar , “Colocalization of Galanin and Luteinizing Hormone‐Releasing Hormone in a Subset of Preoptic Hypothalamic Neurons: Anatomical and Functional Correlates,” Proceedings of the National Academy of Sciences *of the United States of America* 87 (1990): 6326–6330, 10.1073/pnas.87.16.6326. PMC54526 1696726

[60] K. Pihel , S. Hsieh , J. W. Jorgenson , and R. M. Wightman , “Quantal Corelease of Histamine and 5‐Hydroxytryptamine From Mast Cells and the Effects of Prior Incubation,” Biochemistry 37 (1998): 1046–1052, 10.1021/bi9714868. 9454595

[61] C. P. Grabner , S. D. Price , A. Lysakowski , A. L. Cahill , and A. P. Fox , “Regulation of Large Dense‐Core Vesicle Volume and Neurotransmitter Content Mediated by Adaptor Protein 3,” Proceedings of the National Academy of Sciences *of the United States of America* 103 (2006): 10035–10040, 10.1073/pnas.0509844103. PMC1502501 16788073

[62] Z. Zhang and M. B. Jackson , “Membrane Bending Energy and Fusion Pore Kinetics in Ca2+‐Triggered Exocytosis,” Biophysical Journal 98 (2010): 2524–2534, 10.1016/j.bpj.2010.02.043. 20513396 PMC2877347

[63] L. W. Gong , G. A. de Toledo , and M. Lindau , “Exocytotic Catecholamine Release Is Not Associated With Cation Flux Through Channels in the Vesicle Membrane but Na+ Influx Through the Fusion Pore,” Nature Cell Biology 9 (2007): 915–922, 10.1038/ncb1617. 17643118 PMC2871335

[64] C. Amatore , S. Arbault , I. Bonifas , et al., “Correlation Between Vesicle Quantal Size and Fusion Pore Release in Chromaffin Cell Exocytosis,” Biophysical Journal 88 (2005): 4411–4420, 10.1529/biophysj.104.053736. 15792983 PMC1305668

[65] G. T. van Kempen , H. T. vanderLeest , R. J. van den Berg , P. Eilers , and R. H. Westerink , “Three Distinct Modes of Exocytosis Revealed by Amperometry in Neuroendocrine Cells,” Biophysical Journal 100 (2011): 968–977, 10.1016/j.bpj.2011.01.010. 21320441 PMC3037570

[66] N. Vardjan , J. Jorgačevski , M. Stenovec , M. Kreft , and R. Zorec , “Compound Exocytosis in Pituitary Cells,” Annals of the New York Academy of Sciences 1152 (2009): 63–75, 10.1111/j.1749-6632.2008.04008.x. 19161377

[67] J. Nikolaus , K. Hancock , M. Tsemperouli , D. Baddeley , and E. Karatekin , “Optimal Detection of Fusion Pore Dynamics Using Polarized Total Internal Reflection Fluorescence Microscopy,” Frontiers in Molecular Biosciences 8 (2021): 740408, 10.3389/fmolb.2021.740408. 34859048 PMC8631473

[68] H. Bao , D. Das , N. A. Courtney , et al., “Dynamics and Number of Trans‐SNARE Complexes Determine Nascent Fusion Pore Properties,” Nature 554 (2018): 260–263, 10.1038/nature25481. 29420480 PMC5808578

[69] D. E. Chandler and J. E. Heuser , “Arrest of Membrane Fusion Events in Mast Cells by Quick‐Freezing,” The Journal of Cell Biology 86 (1980): 666–674, 10.1083/jcb.86.2.666. 7400221 PMC2111488

[70] B. Satir , C. Schooley , and P. Satir , “Membrane Fusion in a Model System,” The Journal of Cell Biology 56 (1973): 153–176, 10.1083/jcb.56.1.153. 4629881 PMC2108847

[71] W. Schmidt , A. Patzak , G. Lingg , H. Winkler , and H. Plattner , “Membrane Events in Adrenal Chromaffin Cells During Exocytosis: A Freeze‐Etching Analysis After Rapid Cryofixation,” European Journal of Cell Biology 32 (1983): 31–37. 6667695

[72] L. J. Breckenridge and W. Almers , “Currents Through the Fusion Pore That Forms During Exocytosis of a Secretory Vesicle,” Nature 328 (1987): 814–817, 10.1038/328814a0. 2442614

[73] K. Lollike , N. Borregaard , and M. Lindau , “The Exocytotic Fusion Pore of Small Granules Has a Conductance Similar to an Ion Channel,” The Journal of Cell Biology 129 (1995): 99–104, 10.1083/jcb.129.1.99. 7535305 PMC2120381

[74] A. E. Spruce , A. Iwata , and W. Almers , “The First Milliseconds of the Pore Formed by a Fusogenic Viral Envelope Protein During Membrane Fusion,” Proceedings of the National Academy of Sciences *of the United States of America* 88 (1991): 3623–3627, 10.1073/pnas.88.9.3623. PMC51504 2023911

[75] J. Hartmann and M. Lindau , “A Novel Ca 2+‐Dependent Step in Exocytosis Subsequent to Vesicle Fusion,” FEBS Letters 363 (1995): 217–220, 10.1016/0014-5793(95)00318-4. 7737405

[76] M. Lanzrein , N. Käsermann , R. Weingart , and C. Kempf , “Early Events of Semliki Forest Virus‐Induced Cell‐Cell Fusion,” Virology 196 (1993): 541–547, 10.1006/viro.1993.1509. 8372433

[77] E. Karatekin , “Toward a Unified Picture of the Exocytotic Fusion Pore,” FEBS Letters 592 (2018): 3563–3585, 10.1002/1873-3468.13270. 30317539 PMC6353554

[78] G. Dernick , L.‐W. Gong , L. Tabares , G. A. De Toledo , and M. Lindau , “Patch Amperometry: High‐Resolution Measurements of Single‐Vesicle Fusion and Release,” Nature Methods 2 (2005): 699–708, 10.1038/nmeth0905-699. 16118641

[79] L. V. Chernomordik and M. M. Kozlov , “Mechanics of Membrane Fusion,” Nature Structural & Molecular Biology 15 (2008): 675–683, 10.1038/nsmb.1455. PMC2548310 18596814

[80] C. W. Chang , C. W. Chiang , J. D. Gaffaney , E. R. Chapman , and M. B. Jackson , “Lipid‐Anchored Synaptobrevin Provides Little or No Support for Exocytosis or Liposome Fusion,” Journal of Biological Chemistry 291 (2016): 2848–2857, 10.1074/jbc.M115.701169. 26663078 PMC4742749

[81] Q. Fang , K. Berberian , L.‐W. Gong , I. Hafez , J. B. Sørensen , and M. Lindau , “The Role of the C Terminus of the SNARE Protein SNAP‐25 in Fusion Pore Opening and a Model for Fusion Pore Mechanics,” Proceedings of the National Academy of Sciences *of the United States of America* 105 (2008): 15388–15392, 10.1073/pnas.0805377105. PMC2563113 18829435

[82] H. Bao , M. Goldschen‐Ohm , P. Jeggle , B. Chanda , J. M. Edwardson , and E. R. Chapman , “Exocytotic Fusion Pores Are Composed of Both Lipids and Proteins,” Nature Structural & Molecular Biology 23 (2016): 67–73, 10.1038/nsmb.3141. PMC4756907 26656855

[83] S. Sharma and M. Lindau , “Molecular Mechanism of Fusion Pore Formation Driven by the Neuronal SNARE Complex,” Proceedings of the National Academy of Sciences *of the United States of America* 115 (2018): 12751–12756, 10.1073/pnas.1816495115. PMC6294955 30482862

[84] J. Zimmerberg , “How Can Proteolipids Be Central Players in Membrane Fusion?” Trends in Cell Biology 11 (2001): 233–235, 10.1016/s0962-8924(01)02003-7. 11356343

[85] L. V. Chernomordik , G. B. Melikyan , and Y. A. Chizmadzhev , “Biomembrane Fusion: A New Concept Derived From Model Studies Using Two Interacting Planar Lipid Bilayers,” Biochimica et Biophysica Acta (BBA)—Reviews on Biomembranes 906 (1987): 309–352, 10.1016/0304-4157(87)90016-5. 3307918

[86] S. C. Collins , H. W. Do , B. Hastoy , et al., “Increased Expression of the Diabetes Gene SOX4 Reduces Insulin Secretion by Impaired Fusion Pore Expansion,” Diabetes 65 (2016): 1952–1961, 10.2337/db15-1489. 26993066 PMC4996324

[87] A. Gurunian and D. A. Dean , “Multiple Conductance States of Lipid Pores During Voltage‐Clamp Electroporation,” Bioelectrochemistry 151 (2023): 108396, 10.1016/j.bioelechem.2023.108396. 36805203 PMC10040435

[88] K. Debus and M. Lindau , “Resolution of Patch Capacitance Recordings and of Fusion Pore Conductances in Small Vesicles,” Biophysical Journal 78 (2000): 2983–2997, 10.1016/S0006-3495(00)76837-8. 10827977 PMC1300882

[89] S. Sharma and M. Lindau , “The Fusion Pore, 60 Years After the First Cartoon,” FEBS Letters 592 (2018): 3542–3562, 10.1002/1873-3468.13160. 29904915 PMC6231997

[90] W. Shin , L. Ge , G. Arpino , et al., “Visualization of Membrane Pore in Live Cells Reveals a Dynamic‐Pore Theory Governing Fusion and Endocytosis,” Cell 173 (2018): 934–945.e12, 10.1016/j.cell.2018.02.062. 29606354 PMC5935532

[91] T. Weber , B. V. Zemelman , J. A. Mcnew , et al., “SNAREpins: Minimal Machinery for Membrane Fusion,” Cell 92 (1998): 759–772, 10.1016/s0092-8674(00)81404-x. 9529252

[92] R. Hosono , S. Hekimi , Y. Kamiya , et al., “The unc‐18 Gene Encodes a Novel Protein Affecting the Kinetics of Acetylcholine Metabolism in the Nematode *Caenorhabditis elegans* ,” Journal of Neurochemistry 58 (1992): 1517–1525, 10.1111/j.1471-4159.1992.tb11373.x. 1347782

[93] Y. Hata , C. A. Slaughter , and T. C. Südhof , “Synaptic Vesicle Fusion Complex Contains unc‐18 Homologue Bound to Syntaxin,” Nature 366 (1993): 347–351, 10.1038/366347a0. 8247129

[94] T. Shu , H. Jin , J. E. Rothman , and Y. Zhang , “Munc13‐1 MUN Domain and Munc18‐1 Cooperatively Chaperone SNARE Assembly Through a Tetrameric Complex,” Proceedings of the National Academy of Sciences *of the United States of America* 117 (2020): 1036–1041, 10.1073/pnas.1914361117. PMC6969487 31888993

[95] X. Wang , J. Gong , L. Zhu , et al., “Munc13 Activates the Munc18‐1/Syntaxin‐1 Complex and Enables Munc18‐1 to Prime SNARE Assembly,” The EMBO Journal 39 (2020): 103631, doi: 10.15252/embj.2019103631. PMC7429736 32643828

[96] S. Wang , Y. Li , J. Gong , et al., “Munc18 and Munc13 Serve as a Functional Template to Orchestrate Neuronal SNARE Complex Assembly,” Nature Communications 10 (2019): 69, 10.1038/s41467-018-08028-6. PMC6325239 30622273

[97] X. Han , C. T. Wang , J. Bai , E. R. Chapman , and M. B. Jackson , “Transmembrane Segments of Syntaxin Line the Fusion Pore of Ca2+‐Triggered Exocytosis,” Science 304 (2004): 289–292, 10.1126/science.1095801. 15016962

[98] X. Han and M. B. Jackson , “Structural Transitions in the Synaptic SNARE Complex During Ca2+‐Triggered Exocytosis,” The Journal of Cell Biology 172 (2006): 281–293, 10.1083/jcb.200510012. 16418536 PMC2063557

[99] R. E. Guzman , Y. N. Schwarz , J. Rettig , and D. Bruns , “SNARE Force Synchronizes Synaptic Vesicle Fusion and Controls the Kinetics of Quantal Synaptic Transmission,” The Journal of Neuroscience 30 (2010): 10272–10281, 10.1523/JNEUROSCI.1551-10.2010. 20685972 PMC6634679

[100] C. W. Chiang , W. C. Shu , J. Wan , B. A. Weaver , and M. B. Jackson , “Recordings From Neuron–HEK Cell Cocultures Reveal the Determinants of Miniature Excitatory Postsynaptic Currents,” Journal of General Physiology 153 (2021): e202012849, 10.1085/jgp.202012849. PMC7992392 33755721

[101] T. Xu , B. Rammner , M. Margittai , A. R. Artalejo , E. Neher , and R. Jahn , “Inhibition of SNARE Complex Assembly Differentially Affects Kinetic Components of Exocytosis,” Cell 99 (1999): 713–722, 10.1016/s0092-8674(00)81669-4. 10619425

[102] C.‐W. Chang , E. Hui , J. Bai , D. Bruns , E. R. Chapman , and M. B. Jackson , “A Structural Role for the Synaptobrevin 2 Transmembrane Domain in Dense‐Core Vesicle Fusion Pores,” The Journal of Neuroscience 35 (2015): 5772–5780, 10.1523/JNEUROSCI.3983-14.2015. 25855187 PMC4388931

[103] R. Mohrmann , H. De Wit , M. Verhage , E. Neher , and J. B. Sørensen , “Fast Vesicle Fusion in Living Cells Requires at Least Three SNARE Complexes,” Science 330 (2010): 502–505, 10.1126/science.1193134. 20847232

[104] G. van den Bogaart and R. Jahn , “Counting the SNAREs Needed for Membrane Fusion,” Journal of Molecular Cell Biology 3 (2011): 204–205, 10.1093/jmcb/mjr004. 21525018

[105] J. M. Hernandez , A. J. Kreutzberger , V. Kiessling , L. K. Tamm , and R. Jahn , “Variable Cooperativity in SNARE‐Mediated Membrane Fusion,” Proceedings of the National Academy of Sciences *of the United States of America* 111 (2014): 12037–12042, 10.1073/pnas.1407435111. PMC4143004 25092301

[106] C. G. Giraudo , C. Hu , D. You , et al., “SNAREs Can Promote Complete Fusion and Hemifusion as Alternative Outcomes,” The Journal of Cell Biology 170 (2005): 249–260, 10.1083/jcb.200501093. 16027221 PMC2171417

[107] D. Das , H. Bao , K. C. Courtney , L. Wu , and E. R. Chapman , “Resolving Kinetic Intermediates During the Regulated Assembly and Disassembly of Fusion Pores,” Nature Communications 11 (2020): 231, 10.1038/s41467-019-14072-7. PMC6957489 31932584

[108] C.‐T. Wang , R. Grishanin , C. A. Earles , et al., “Synaptotagmin Modulation of Fusion Pore Kinetics in Regulated Exocytosis of Dense‐Core Vesicles,” Science 294 (2001): 1111–1115, 10.1126/science.1064002. 11691996

[109] H. Park , Y. Li , and R. W. Tsien , “Influence of Synaptic Vesicle Position on Release Probability and Exocytotic Fusion Mode,” Science 335 (2012): 1362–1366, 10.1126/science.1216937. 22345401 PMC3776413

[110] K. Stiasny and F. X. Heinz , “Effect of Membrane Curvature‐Modifying Lipids on Membrane Fusion by Tick‐Borne Encephalitis Virus,” Journal of Virology 78 (2004): 8536–8542, 10.1128/JVI.78.16.8536-8542.2004. 15280462 PMC479076

[111] R. B. Lira , J. C. F. Hammond , R. R. M. Cavalcanti , M. Rous , K. A. Riske , and W. H. Roos , “The Underlying Mechanical Properties of Membranes Tune Their Ability to Fuse,” Journal of Biological Chemistry 299 (2023): 105430, 10.1016/j.jbc.2023.105430. 37926280 PMC10716014

[112] S. Haldar , E. Mekhedov , C. D. McCormick , P. S. Blank , and J. Zimmerberg , “Lipid‐Dependence of Target Membrane Stability During Influenza Viral Fusion,” Journal of Cell Science 132 (2018): jcs218321, 10.1242/jcs.218321. PMC6398481 29967032

[113] M. M. Kozlov and L. V. Chernomordik , “Membrane Tension and Membrane Fusion,” Current Opinion in Structural Biology 33 (2015): 61–67, 10.1016/j.sbi.2015.07.010. 26282924 PMC4641764

[114] L. Wu , K. C. Courtney , and E. R. Chapman , “Cholesterol Stabilizes Recombinant Exocytic Fusion Pores by Altering Membrane Bending Rigidity,” Biophysical Journal 120 (2021): 1367–1377, 10.1016/j.bpj.2021.02.005. 33582136 PMC8105710

[115] S. Biswas , S. R. Yin , P. S. Blank , and J. Zimmerberg , “Cholesterol Promotes Hemifusion and Pore Widening in Membrane Fusion Induced by Influenza Hemagglutinin,” The Journal of General Physiology 131 (2008): 503–513, 10.1085/jgp.200709932. 18443361 PMC2346574

[116] A. Linetti , A. Fratangeli , E. Taverna , et al., “Cholesterol Reduction Impairs Exocytosis of Synaptic Vesicles,” Journal of Cell Science 123 (2010): 595–605, 10.1242/jcs.060681. 20103534

[117] S. Koseoglu , S. A. Love , and C. L. Haynes , “Cholesterol Effects on Vesicle Pools in Chromaffin Cells Revealed by Carbon‐Fiber Microelectrode Amperometry,” Analytical and Bioanalytical Chemistry 400 (2011): 2963–2971, 10.1007/s00216-011-5002-7. 21523329

[118] B. Rituper , A. Guček , M. Lisjak , et al., “Vesicle Cholesterol Controls Exocytotic Fusion Pore,” Cell Calcium 101 (2022): 102503, 10.1016/j.ceca.2021.102503. 34844123

[119] E. Tanguy , P. Costé De Bagneaux , N. Kassas , et al., “Mono‐ and Poly‐Unsaturated Phosphatidic Acid Regulate Distinct Steps of Regulated Exocytosis in Neuroendocrine Cells,” Cell Reports 32 (2020): 108026, 10.1016/j.celrep.2020.108026. 32814056

[120] B. S. Stratton , J. M. Warner , Z. Wu , et al., “Cholesterol Increases the Openness of SNARE‐Mediated Flickering Fusion Pores,” Biophysical Journal 110 (2016): 1538–1550, 10.1016/j.bpj.2016.02.019. 27074679 PMC4833774

[121] H. Y. Ali Moussa , K. C. Shin , J. Ponraj , et al., “Requirement of Cholesterol for Calcium‐Dependent Vesicle Fusion by Strengthening Synaptotagmin‐1‐Induced Membrane Bending,” Advanced Science 10 (2023): 2206823, 10.1002/advs.202206823. 37058136 PMC10214243

[122] M. Omar‐Hmeadi , A. Guček , and S. Barg , “Local PI(4,5)P2 Signaling Inhibits Fusion Pore Expansion During Exocytosis,” Cell Reports 42 (2023): 112036, 10.1016/j.celrep.2023.112036. 36701234

[123] A. Makam , J. Wahlund , N. R. Gandasi , and A. Hatamie , “Single‐Vesicle Microelectroanalysis Reveals the Role of PIP2 Phospholipid in Vesicle Opening Dynamics and Its Potential Role in Exocytosis,” ACS Omega 10 (2025): 18889–18898, 10.1021/acsomega.5c00864. 40385141 PMC12079234

[124] B. R. Bhaskar , L. Yadav , M. Sriram , et al., “Differential SNARE Chaperoning by Munc13‐1 and Munc18‐1 Dictates Fusion Pore Fate at the Release Site,” Nature Communications 15 (2024): 4132, 10.1038/s41467-024-46965-7. PMC11099066 38755165

[125] L. Chernomordik , A. Chanturiya , J. Green , and J. Zimmerberg , “The Hemifusion Intermediate and Its Conversion to Complete Fusion: Regulation by Membrane Composition,” Biophysical Journal 69 (1995): 922–929, 10.1016/S0006-3495(95)79966-0. 8519992 PMC1236321

[126] Z. Zhang , E. Hui , E. R. Chapman , and M. B. Jackson , “Phosphatidylserine Regulation of Ca2+‐Triggered Exocytosis and Fusion Pores in PC12 Cells,” Molecular Biology of the Cell 20 (2009): 5086–5095, 10.1091/mbc.e09-08-0691. 19828732 PMC2793286

[127] A. J. B. Kreutzberger , V. Kiessling , B. Liang , S.‐T. Yang , J. D. Castle , and L. K. Tamm , “Asymmetric Phosphatidylethanolamine Distribution Controls Fusion Pore Lifetime and Probability,” Biophysical Journal 113 (2017): 1912–1915, 10.1016/j.bpj.2017.09.014. 29037600 PMC5685784

[128] J.‐S. Rhee , A. Betz , S. Pyott , et al., “β Phorbol Ester‐ and Diacylglycerol‐Induced Augmentation of Transmitter Release Is Mediated by Munc13s and Not by PKCs,” Cell 108 (2002): 121–133, 10.1016/s0092-8674(01)00635-3. 11792326

[129] D. Araç , X. Chen , H. A. Khant , et al., “Close Membrane‐Membrane Proximity Induced by Ca2+‐Dependent Multivalent Binding of Synaptotagmin‐1 to Phospholipids,” Nature Structural & Molecular Biology 13 (2006): 209–217, 10.1038/nsmb1056. 16491093

[130] G. Van Den Bogaart , K. Meyenberg , H. J. Risselada , et al., “Membrane Protein Sequestering by Ionic Protein–Lipid Interactions,” Nature 479 (2011): 552–555, 10.1038/nature10545. 22020284 PMC3409895

[131] T. Söllner , S. W. Whiteheart , M. Brunner , et al., “SNAP Receptors Implicated in Vesicle Targeting and Fusion,” Nature 362 (1993): 318–324, 10.1038/362318a0. 8455717

[132] I. Dulubova , S. Sugita , S. Hill , et al., “A Conformational Switch in Syntaxin During Exocytosis: Role of munc18,” The EMBO Journal 18 (1999): 4372–4382, 10.1093/emboj/18.16.4372. 10449403 PMC1171512

[133] J. Jiao , M. He , S. A. Port , et al., “Munc18‐1 Catalyzes Neuronal SNARE Assembly by Templating SNARE Association,” eLife 7 (2018): e41771, 10.7554/eLife.41771. PMC6320071 30540253

[134] M. Camacho , B. Quade , T. Trimbuch , et al., “Control of Neurotransmitter Release by Two Distinct Membrane‐Binding Faces of the Munc13‐1 C1C2B Region,” eLife 10 (2021): 72030, 10.7554/eLife.72030. PMC8648301 34779770

[135] R. J. Fisher , J. Pevsner , and R. D. Burgoyne , “Control of Fusion Pore Dynamics During Exocytosis by Munc18,” Science 291 (2001): 875–878, 10.1126/science.291.5505.875. 11157167

[136] A. Gulyás‐Kovács , H. De Wit , I. Milosevic , et al., “Munc18‐1: Sequential Interactions With the Fusion Machinery Stimulate Vesicle Docking and Priming,” The Journal of Neuroscience 27 (2007): 8676–8686, 10.1523/JNEUROSCI.0658-07.2007. 17687045 PMC6672934

[137] L. F. Ciufo , J. W. Barclay , R. D. Burgoyne , and A. Morgan , “Munc18‐1 Regulates Early and Late Stages of Exocytosis via Syntaxin‐Independent Protein Interactions,” Molecular Biology of the Cell 16 (2005): 470–482, 10.1091/mbc.e04-08-0685. 15563604 PMC545880

[138] J. Jorgacevski , M. Potokar , S. Grilc , et al., “Munc18‐1 Tuning of Vesicle Merger and Fusion Pore Properties,” Journal of Neuroscience 31 (2011): 9055–9066, 10.1523/JNEUROSCI.0185-11.2011. 21677188 PMC6622955

[139] J. Rizo and J. Xu , “The Synaptic Vesicle Release Machinery,” Annual Review of Biophysics 44 (2015): 339–367, 10.1146/annurev-biophys-060414-034057. 26098518

[140] I. Stefani , J. Iwaszkiewicz , and D. Fasshauer , “Exploring the Conformational Changes of the Munc18‐1/Syntaxin 1a Complex,” Protein Science 33 (2023): 4870, 10.1002/pro.4870. PMC10895456 38109275

[141] M. N. Wu , T. Fergestad , T. E. Lloyd , Y. He , K. Broadie , and H. J. Bellen , “Syntaxin 1A Interacts With Multiple Exocytic Proteins to Regulate Neurotransmitter Release In Vivo,” Neuron 23 (1999): 593–605, 10.1016/s0896-6273(00)80811-9. 10433270

[142] E. A. Fon and R. H. Edwards , “Molecular Mechanisms of Neurotransmitter Release,” Muscle & Nerve 24 (2001): 581–601, 10.1002/mus.1044. 11317268

[143] N.‐R. Bin , C. H. Jung , B. Kim , et al., “Chaperoning of Closed Syntaxin‐3 Through Lys46 and Glu59 in Domain 1 of Munc18 Proteins Is Indispensable for Mast Cell Exocytosis,” Journal of Cell Science 128 (2015): 1946–1960, 10.1242/jcs.165662. 25795302

[144] C. Morey , C. N. Kienle , T. H. Klöpper , P. Burkhardt , and D. Fasshauer , “Evidence for a Conserved Inhibitory Binding Mode Between the Membrane Fusion Assembly Factors Munc18 and Syntaxin in Animals,” Journal of Biological Chemistry 292 (2017): 20449–20460, 10.1074/jbc.M117.811182. 29046354 PMC5733584

[145] M. E. Graham , J. W. Barclay , and R. D. Burgoyne , “Syntaxin/Munc18 Interactions in the Late Events During Vesicle Fusion and Release in Exocytosis,” Journal of Biological Chemistry 279 (2004): 32751–32760, 10.1074/jbc.M400827200. 15175344

[146] R. G. Staal , E. V. Mosharov , and D. Sulzer , “Dopamine Neurons Release Transmitter via a Flickering Fusion Pore,” Nature Neuroscience 7 (2004): 341–346, 10.1038/nn1205. 14990933

[147] S. Scepek , “Fusion Pore Expansion in Horse Eosinophils Is Modulated by Ca2+ and Protein Kinase C via Distinct Mechanisms,” The EMBO Journal 17 (1998): 4340–4345, 10.1093/emboj/17.15.4340. 9687502 PMC1170767

[148] X. Lou , N. Korogod , N. Brose , and R. Schneggenburger , “Phorbol Esters Modulate Spontaneous and Ca2+‐Evoked Transmitter Release via Acting on Both Munc13 and Protein Kinase C,” The Journal of Neuroscience 28 (2008): 8257–8267, 10.1523/JNEUROSCI.0550-08.2008. 18701688 PMC6670566

[149] A. Betz , U. Ashery , M. Rickmann , et al., “Munc13‐1 Is a Presynaptic Phorbol Ester Receptor That Enhances Neurotransmitter Release,” Neuron 21 (1998): 123–136, 10.1016/s0896-6273(00)80520-6. 9697857

[150] C. Mitine , A. Dutreix , and E. van der Schueren , “Tangential Breast Irradiation: Influence of Technique of Set‐Up on Transfer Errors and Reproducibility,” Radiotherapy and Oncology 22 (1991): 308–310, 10.1016/0167-8140(91)90168-g. 1792326

[151] R. S. Redman , T. J. Searl , J. K. Hirsh , and E. M. Silinsky , “Opposing Effects of Phorbol Esters on Transmitter Release and Calcium Currents at Frog Motor Nerve Endings,” The Journal of Physiology 501, no. pt. 1 (1997): 41–48, 10.1111/j.1469-7793.1997.041bo.x. 9174992 PMC1159502

[152] C. N. Scholfield and A. J. Smith , “A Phorbol Diester‐Induced Enhancement of Synaptic Transmission in Olfactory Cortex,” British Journal of Pharmacology 98 (1989): 1344–1350, 10.1111/j.1476-5381.1989.tb12683.x. 2558761 PMC1854809

[153] T. Hori , Y. Takai , and T. Takahashi , “Presynaptic Mechanism for Phorbol Ester‐Induced Synaptic Potentiation,” The Journal of Neuroscience 19 (1999): 7262–7267, 10.1523/JNEUROSCI.19-17-07262.1999. 10460232 PMC6782531

[154] R. S. Zucker , “Minis: Whence and Wherefore?” Neuron 45 (2005): 482–484, 10.1016/j.neuron.2005.02.003. 15721234

[155] U. Blank , I. K. Madera‐Salcedo , L. Danelli , et al., “Vesicular Trafficking and Signaling for Cytokine and Chemokine Secretion in Mast Cells,” Frontiers in Immunology 5 (2014): 453, 10.3389/fimmu.2014.00453. 25295038 PMC4170139

[156] R. Tsutsumi and N. J. Webster , “GnRH Pulsatility, the Pituitary Response and Reproductive Dysfunction,” Endocrine Journal 56 (2009): 729–737, 10.1507/endocrj.k09e-185. 19609045 PMC4307809

[157] J. D. Veldhuis , “Mechanisms and Biological Significance of Pulsatile Hormone Secretion. Symposium Proceedings. London, United Kingdom, 2–4 March 1999,” Novartis Foundation Symposium 227 (2000): 1–4. 10858070

[158] J. L. Christian , “Morphogen Gradients in Development: From Form to Function,” WIREs Developmental Biology 1 (2012): 3–15, 10.1002/wdev.2. 23801664 PMC3957335

[159] H. Huang , S. Liu , and T. B. Kornberg , “Glutamate Signaling at Cytoneme Synapses,” Science 363 (2019): 948–955, 10.1126/science.aat5053. 30819957 PMC7008667

[160] L. Shindarov , A. Galabov , G. Mitev , P. Tekerlekov , N. Neykova , and G. Vassilev , “Antiviral Effect of Thiourea and Urea Derivatives in Experimental Foot‐and‐Mouth Disease,” Zentralblatt Für Veterinärmedizin Reihe B 20 (1973): 111–117, 10.1111/j.1439-0450.1973.tb01108.x. 4722357

[161] R. Malinow and R. W. Tsien , “Presynaptic Enhancement Shown by Whole‐Cell Recordings of Long‐Term Potentiation in Hippocampal Slices,” Nature 346 (1990): 177–180, 10.1038/346177a0. 2164158

[162] S. Choi , J. Klingauf , and R. W. Tsien , “Postfusional Regulation of Cleft Glutamate Concentration During LTP at ‘Silent Synapses’,” Nature Neuroscience 3 (2000): 330–336, 10.1038/73895. 10725921

[163] S. H. Oliet , R. C. Malenka , and R. A. Nicoll , “Bidirectional Control of Quantal Size by Synaptic Activity in the Hippocampus,” Science 271 (1996): 1294–1297, 10.1126/science.271.5253.1294. 8638114

[164] D. M. Kullmann and R. A. Nicoll , “Long‐Term Potentiation Is Associated With Increases in Quantal Content and Quantal Amplitude,” Nature 357 (1992): 240–244, 10.1038/357240a0. 1317014

[165] J. J. Renger , C. Egles , and G. Liu , “A Developmental Switch in Neurotransmitter Flux Enhances Synaptic Efficacy by Affecting AMPA Receptor Activation,” Neuron 29 (2001): 469–484, 10.1016/s0896-6273(01)00219-7. 11239436

[166] S. Choi , J. Klingauf , and R. W. Tsien , “Fusion Pore Modulation as a Presynaptic Mechanism Contributing to Expression of Long‐Term Potentiation,” Philosophical Transactions of the Royal Society of London Series B: Biological Sciences 358 (2003): 695–705, 10.1098/rstb.2002.1249. 12740115 PMC1693158

[167] A. M. Petrov , G. F. Zakirjanova , I. V. Kovyazina , A. N. Tsentsevitsky , and E. A. Bukharaeva , “Adrenergic Receptors Control Frequency‐Dependent Switching of the Exocytosis Mode Between “Full‐Collapse” and “Kiss‐and‐Run” in Murine Motor Nerve Terminal,” Life Sciences 296 (2022): 120433, 10.1016/j.lfs.2022.120433. 35219696

[168] X. M. Shen , D. Selcen , J. Brengman , and A. G. Engel , “Mutant SNAP25B Causes Myasthenia, Cortical Hyperexcitability, Ataxia, and Intellectual Disability,” Neurology 83 (2014): 2247–2255, 10.1212/WNL.0000000000001079. 25381298 PMC4277673

